# Excessive fructose intake inhibits skeletal development in adolescent rats *via* gut microbiota and energy metabolism

**DOI:** 10.3389/fmicb.2022.952892

**Published:** 2022-09-14

**Authors:** Tianlin Gao, Chunyan Tian, Ge Tian, Li Ma, Lili Xu, Wendong Liu, Jing Cai, Feng Zhong, Huaqi Zhang, Aiguo Ma

**Affiliations:** ^1^School of Public Health, Qingdao University, Qingdao, China; ^2^Institute of Nutrition and Health, Qingdao University, Qingdao, China; ^3^Department of Nutrition, The Affiliated Hospital of Qingdao University, Qingdao, China; ^4^Department of Endocrinology and Metabolism, The Affiliated Hospital of Qingdao University, Qingdao, China; ^5^Department of Pediatrics, Qingdao Municipal Hospital, Affiliated to Qingdao University, Qingdao, China

**Keywords:** fructose, bone development, gut microbiota, energy metabolism, adolescent

## Abstract

Excessive fructose intake from desserts and beverages may influence bone development among adolescents. The gut microbiota (GM) and energy metabolism play important roles in bone development. In this study, 40 female adolescent rats were randomly assigned to the control group, the fructose group with two concentrations, and the glucose group as the positive control group. After 10 weeks, serum glucose and lipids were detected by means of an automatic analyzer, and the bone microstructure was analyzed by Micro-CT. Then, the GM was determined *via* 16S rRNA sequencing analysis, and energy metabolism was detected by measuring serum carbohydrate metabolites. At last, bone metabolism markers were measured *via* ELISA kits. The results showed that excessive fructose intake could increase body weight and influence the glucolipid metabolism of female adolescent rats. Meanwhile, the bone microstructures were impaired with excessive fructose intake. Mechanistically, excessive fructose intake shifted the GM of rats with the decrease of *Lachnospiraceae, Ruminococcaceae*, and increase of *Allobaculum, Lachnospiraceae.* Energy metabolism analysis suggested that most metabolites of fructose did not enter the tricarboxylic acid cycle to provide energy for the body’s development. Furthermore, serum bone metabolism markers showed that excessive fructose intake could decrease both bone formation and resorption. Our results suggested that excessive fructose intake could inhibit skeletal development in adolescents. One potential mechanism might be that it affected the intestinal microbiota homeostasis in the juvenile body, thus changing the energy metabolism level, and ultimately affecting the bone metabolic balance.

## Introduction

Fructose is widely used in soft drinks, dairy products, and processed pastries due to its sweetener properties. One of the main consuming groups of sweets is adolescents, who are undergoing crucial body development stages (Hess et al., 2012). Children aged 2–9 years in Europe and minors aged 2–18 years in the United States were reported to intake 14% of their daily energy consumption from free sugars (Poti et al., 2013; Svensson et al., 2014). Since the type of sugar added to beverages has changed from sucrose to fructose corn syrup (HFCS), intake of fructose increased rapidly. Excessive fructose consumption in adolescents might lead to adverse effects, including obesity, insulin resistance, and metabolic syndrome (Williams et al., 2020; Xu and Yu, 2022). Excessive fructose intake in adolescents may also affect bone health as adolescence is the critical period of bone development. Excessive intake of sweetened beverages rich in fructose was reported to reduce bone volume and increase the risks of fractures in children and adolescents (Ma and Jones, 2004; Libuda et al., 2008). However, the independent mechanisms by which fructose acts on bone development and bone metabolism remain to be established.

The mechanism of fructose affecting bone metabolism is a complex process and has not been fully explored. Some reports showed it can be through disturbing the absorption, reabsorption, and excretion of essential vitamins and minerals for healthy bone growth (Ma and Jones, 2004; Tian and Yu, 2017). Fructose could affect vitamin D metabolism and reduce vitamin D-dependent calcium transport in the rodent gut (Douard et al., 2014). However, there are many other factors that could affect bone metabolism. It is of great significance to fully understand the effect of fructose on bone development by exploring the mechanism of how fructose affects bone metabolism. The gut microbiota (GM) co-evolves with the host and plays an important role in maintaining the health of the host (Rolig et al., 2015; Li Y. et al., 2022). The GM has been conformed to influence physiological and pathological processes in distal organs and tissues, including the bone (Feng et al., 2018; Lu et al., 2021). Extensive research has demonstrated that the effects of GM on bone metabolism and perturbation of the GM could drive skeletal deterioration in pathophysiological states (Li et al., 2019). The gut–bone axis is currently the focus of extensive research as a target for exploring the association between food composition and bone metabolism (Hansen and Frost, 2022). Meanwhile, bone metabolism is in a dynamic balance between osteogenic and osteoclastic differentiation over the course of life. Energy metabolism, from glycolysis in the cytosol to oxidative phosphorylation (OXPHOS), is recognized as a metabolic switch of bone metabolism (Zheng et al., 2020). Adolescence is the period when the body’s energy metabolism is the most vigorous. During this period, osteogenic differentiation is stronger than osteoclastic differentiation and finally promotes the rapid development of bone. Fructose, as a kind of carbohydrate, can be involved in the body’s energy metabolism and its excessive intake might more or less alter the energy metabolism and then influence bone metabolic homeostasis. However, there were limited studies that reported the relationship between fructose, GM, energy metabolism, and bone development.

**Scheme 1:**
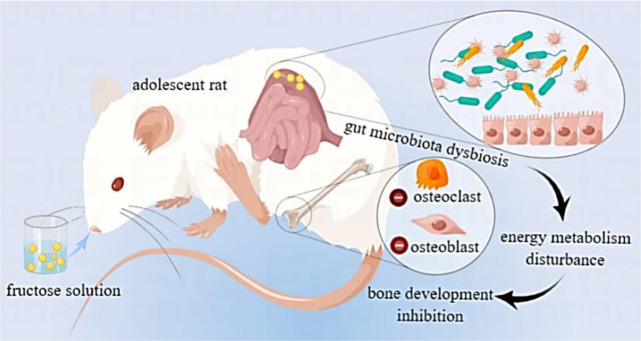
Graphical abstract of excessive fructose intake inhibiting the skeletal development of adolescent rats *via* gut microbiota and energy metabolism.

To clarify whether and how overconsumption of fructose may affect bone development in adolescents, rat models *via* free drink of different concentrations of fructose solution were established in this study (Scheme 1). The bone status was determined by micro-CT. Gut microbiota balance and energy metabolism of rats in different groups were also tested. Since excessive fructose intake can cause additional energy intake glucose was selected as the positive control group in order to better evaluate the special effects of fructose.

## Materials and methods

### Animals

Three-week-old female Sprague-Dawley rats were obtained from Shandong Lukang Laboratory Animal Center (Qingdao, China) and maintained at room temperature (21–23°C), 12 h light cycle, with free access to water and standard chow (AIN-93G, Keaoxieli Fodder, Beijing, China). After 1 week of adaptation, the rats were randomly assigned to the control group (CON, *n* = 10), fructose groups with two concentrations (FRU, 10% and 20% w/vol, *n* = 10), and the glucose group (GLU, 10% w/vol, *n* = 10) as the positive control group. The body weights were measured once a week. All procedures were approved by the Animal Care and Use Committee of the Medical College, Qingdao University (QDU-AEC -2019117) and were performed in accordance with the International Institutes of Health Guidelines for the care and use of laboratory animals. After 10 weeks, animals were euthanized and blood and the left femur were collected for analysis. To analyze the gut microbiome, the colon content was collected into sterile centrifuge tubes using sterile forceps. Then serum, femurs, and colon contents were stored at −80°C until analysis.

### Serum measurements of glucolipid metabolism

Serum glucose, high-density lipoprotein (HDL), low-density lipoprotein (LDL), total cholesterol (TC), and total triglyceride (TG) levels were measured by means of an automatic analyzer (7600, Hitachi, Japan). Serum fructose was determined *via* Fructose Assay Kit (Abcam, United Kingdom).

### Micro-CT analysis

The left femurs were placed in the specimen slot and then scanned by X-ray. The 360-degree scanning of femurs was performed using the following parameters: 80 kV of source voltage, 100 μA of source current, a resolution of 9 μm, and the NRecon software (version 1.6.10.1, Bruker-mCT, Kontich, Belgium) were used for 3D reconstruction. The images were processed by DataViewer software (version 1.5.1.9, Bruker-mCT), and the region of interest was selected using CTAn software (version 1.15.2.2, Bruker-mCT, Belgium). Then to reconstruct the microstructure of cancellous bone and cortical bone, the processed images were imported to Mimics software (version 19.0). Finally, the indicators of bone mineral density (BMD), bone volume fraction (BV/TV), structure model index (SMI), trabecular thickness (Tb.Th), trabecular number (Tb.N), trabecular separation (Tb.Sp), total porosity [Po(tot)], and connectivity density (Con.D) were measured and evaluated by means of the above-mentioned software.

### 16S rRNA sequencing analysis

Total genomic DNA from samples was extracted using the QIAamp Fast DNA Stool Mini Kit (QIAGEN, Hilden, Germany) according to the manufacturer’s instructions. The concentration and purity of DNA were monitored by agarose gel electrophoresis. The V3–V4 variable regions of the 16S ribosomal RNA (rRNA) genes was amplified with primers 343-F and 798-R using polymerase chain reaction (PCR). Then the amplified products were purified using AMPure XP beads and quantified using a Qubit Fluorometer (Invitrogen, Carlsbad, CA, United States).

Sequencing library was constructed using the TruSeqTM DNA PCR-free Sample Prep kit (Illumina, San Diego, CA, United States). The library was quantified and sequenced on a MiSeq PE300 platform (Illumina, MiSeq Reagent Kit v3). The raw reads were merged using Flash software (maximum overlap <200), and the complete paired-end sequences were acquired. Finally, the complete sequences were processed using split _libraries of QIIME software. High-quality reads for bioinformatics analysis were performed, and the nucleotide sequence consistency level of 96% was clustered into operational taxonomic units (OTUs). The sequence with the most frequency in each OTU was chosen as the representative sequence.

### Central carbon metabolism test

The serum samples were defrosted in an ice water bath and then mixed in a vortex for 30 s. A total of 100 μL of aliquot of each individual sample was transferred to an Eppendorf tube. After the addition of equal volumes of extraction buffer (acetonitrile/methanol/water, 2/2/1, v/v/v), the samples were vortexed for 1 min. The samples were stored at −20°C for 2 h and then 100 μL of 0.2% acetic acid in acetonitrile was added. After centrifugation at 12,000 rpm for 15 min at 4°C, 150 μL of supernatant was collected and filtered. Samples were analyzed in Agilent 1290 Infinity II liquid chromatograph-6546 LC/Q-TOF, equipped with Agilent InfinityLab Poroshell 120 HILIC-Z (2.1 × 100 mm, 2.7 μm) column. Mobile phase A was 10 mM ammonium acetate in water, pH = 9, and the mobile phase B was 10 mM ammonium acetate solution: acetonitrile (1/9, v/v), pH = 9. The HPLC program was as follows: 0 min, 0%A; 11.5 min, 30%A; 12 min, 0% A; 20 min 0% A. The column temperature was 30°C, and the injection volume was 5 μL. The mass spectrometry detection was performed with both positive and negative electrospray ionization mode. The MS and MS/MS spectra were obtained in the range of m/z 50–750. The final optimized mass parameters were set as follows: nebulizer gas and heater gas at 40 psig, ion spray voltage at 3000 v, the turbo spray temperature at 300°C, and collision energy at 115 eV.

### Serum measurements of bone metabolism markers

Serum bone metabolism markers of osteocalcin (OCN), bone-specific alkaline phosphatase (BALP), type 1 collagen N-terminal propeptide (P1NP), and tartaric acid alkaline phosphatase (TRACP) were detected by enzyme-linked immunosorbent assay using commercial Elisa kits (Jingkang Bioengineering Company, Shanghai, China), absorbance value at 450 nm on the microplate reader (Thermo, United States).

### Statistical analysis

Statistical analysis was performed using SPSS (version 21.0) and GraphPad Prism (version 9). We used means and standard errors to describe the measurements from the four groups, and one-way analysis of variance (ANOVA) followed by Tukey’s multiple comparison tests to test the statistical significance of differences between groups with *P* < 0.05 considered as statistically significant. For GM analysis, the Chao1, Shannon, and Simpson indices were used to evaluate the α-diversity of microbiota; and the primary component analysis (PCA) was used to explore discrepancies in gut microbiota structure between groups. The LEfSe algorithm was used to show the abundances of dominant microbiota between groups, and the relative abundance analysis was used to reveal the composition of different community structures. The GM analysis was performed using the platform of OE Biotech. Co., Ltd., China.

## Results

### Excessive fructose intake increased body weight

**FIGURE 1:**
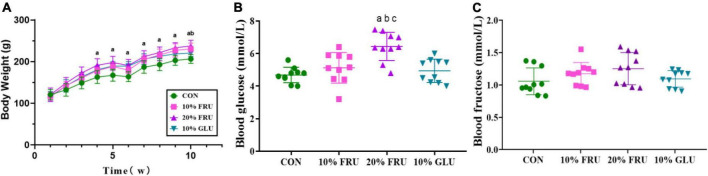
Effects of excessive fructose intake on the physical and physiological parameters of rats: **(A)** body weight, **(B)** blood glucose, and **(C)** blood fructose after 10 weeks. The values were presented as mean ± SD (*n* = 10); a means *p* < 0.05 compared with the CON group; b means *p* < 0.05 compared with the 10% GLU group; and c means *p* < 0.05 compared with the 10% FRU group.

As most sugar-sweetened beverages contained 10% syrups, 10% was chosen as the concentration for the study. To further assess the effect of excessive fructose intake on bone development, a 20% fructose solution was also selected in this study. The body weights of different groups are shown in Figure 1A. At the beginning of the experiment, the body weights were not significantly different between the four groups. The weight of rats in the 20% FRU group was significantly higher than that of the CON group after 4 weeks. After 9 weeks, the weight of rats in 10% FRU and 10% GLU groups was significantly higher than that of the CON group. After 10 weeks, the weight of rats in the 20% FRU group was significantly higher than the 10% GLU group, but not statistically different from that of the 10% FRU group.

### Excessive fructose intake influenced the glucolipid metabolism

The serum glucose and fructose levels are shown in Figures 1B,C. The serum glucose level of the 20% FRU group was significantly higher than those of the other three groups (*p* < 0.05), but no significant differences were found between the CON, 10% FRU, and GLU groups. For the serum fructose level, there were no significant differences between the four groups, and the 20% FRU group showed a trend of higher levels when compared with the CON (*p* = 0.13) and 10% GLU (*p* = 0.28) groups.

Table 1 shows serum high-density lipoprotein (HDL), low-density lipoprotein (LDL), total cholesterol (TC), and total triglyceride (TG) levels. There were no significant differences in LDL-C levels between the four groups. However, serum TG and TC levels in the 20% FRU group were significantly higher than those of the other three groups (*p* < 0.05). It was also observed that the serum HDL-C level in the 10% FRU group was significantly lower than that of the CON group.

**TABLE 1:** Serum concentration of HDL-C, LDL-C, TG, and TC in different groups.

Group	HDL-c (mM)	LDL-c (mM)	TG (mM)	TC (mM)
CON	1.01 ± 0.16	0.51 ± 0.14	0.33 ± 0.10	4.43 ± 0.39
10% FRU	0.74 ± 0.20a	0.43 ± 0.10	0.46 ± 0.10	5.29 ± 0.99
20% FRU	0.77 ± 0.22	0.38 ± 0.14	1.06 ± 0.57abc	7.00 ± 1.75abc
10% GLU	0.93 ± 0.20	0.46 ± 0.07	0.41 ± 0.11	5.04 ± 0.34

The values were presented as mean ± SD (*n* = 10); a means *p* < 0.05 compared with the CON group; b means *p* < 0.05 compared with the 10% GLU group; and c means *p* < 0.05 compared with the 10% FRU group.

### Excessive fructose intake impaired the bone microstructure

**FIGURE 2:**
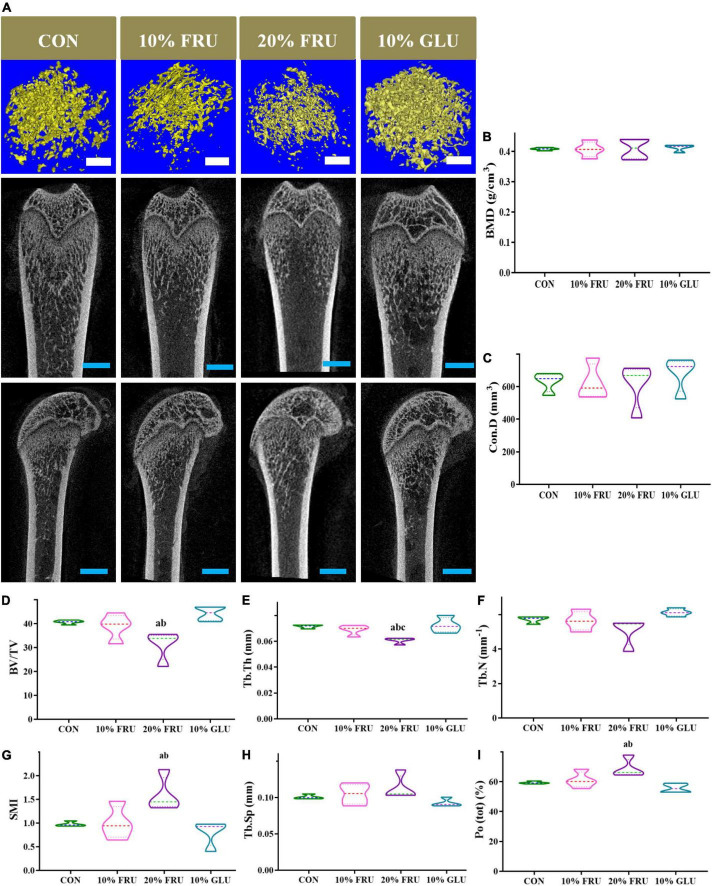
3D reconstruction images and bone microstructure parameters of the metaphysis of distal femur in different groups: **(A)** 3D reconstruction of the metaphyseal of the distal femur and **(B–I)** microstructure parameters of distal femur metaphyseal bone. The values were presented as mean ± SD (*n* = 4); a means *p* < 0.05 compared with the CON group; b means *p* < 0.05 compared with the 10% GLU group; and c means *p* < 0.05 compared with the 10% FRU group.

The indicators of trabecular bone microstructure are shown in Figure 2. BMD and Con.D had no significant variation among the four groups (*p* > 0.05). The BV/TV and Tb.Th of the 20% FRU group were observed significantly lower than those of the CON and 10% GLU groups (*p* < 0.05). Whereas SMI and Po(tot) were significantly higher than those of the CON and 10% GLU groups (*p* < 0.05). No significant differences were found among the CON, 10% FRU, and 10% GLU groups. However, the 10% GLU group showed slightly higher levels of BV/TV and Tb.N, while lower levels of Tb.Sp and Po(tot) compared to the 10% FRU group.

### Excessive fructose intake changed the balance of bacterial gut microbiota

**FIGURE 3:**
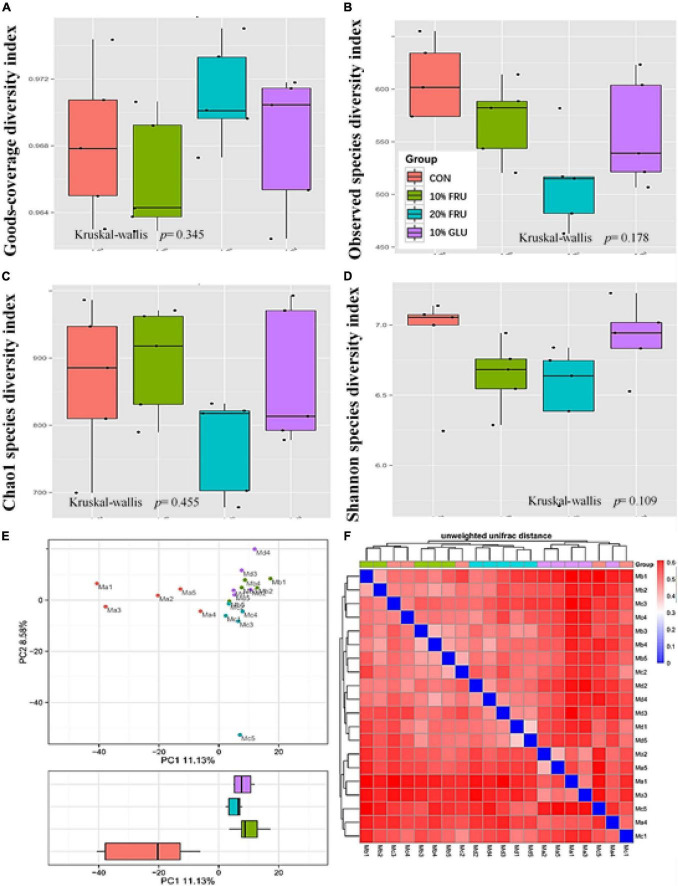
Responses of the diversity, richness, and structure of the gut microbiota to fructose treatment in rats: **(A)** goods-coverage diversity index, **(B)** observed species diversity index, **(C)** Chao1 index, **(D)** Shannon index, **(E)** principal component analysis (PCA) score plot based on weights, and **(F)** unweighted UniFrac distance. Values were presented as mean ± SEM (*n* = 5). Ma or red color represented the CON group, Mb or green color represented the 10% FRU group, Mc or blue color represented the 20% FRU group, and Md or purple color represented the 10% GLU group.

**FIGURE 4:**
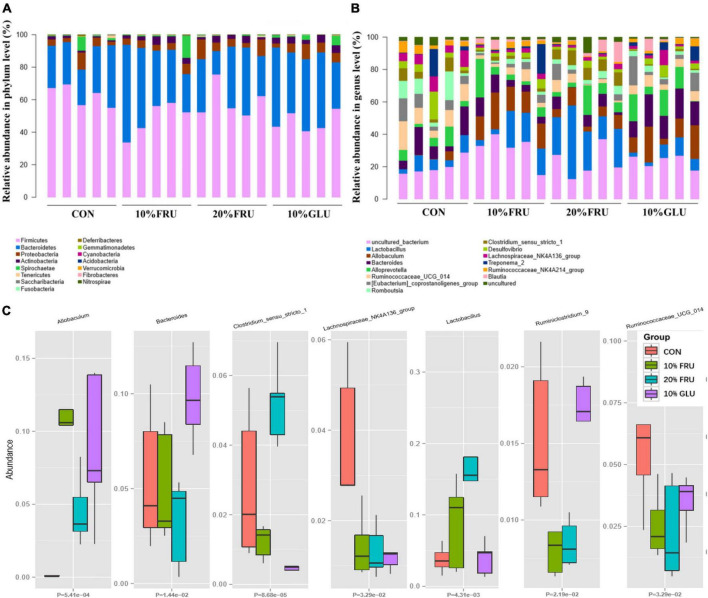
Relative abundances of the gut microbiota at the phylum level **(A)**, genus level **(B)** and some different bacterial taxa (*Allobaculum, Bacteroides, Clostridium, Lachnospiraceae, Lactobacillales*, and *Ruminococcaceae*) obtained in fecal microbiota from the LefSe results **(C)**. Values were presented as mean ± SEM (*n* = 5). Red color represented the CON group, green color represented the 10% FRU group, blue color represented the 20% FRU group, and purple color represented the 10% GLU group.

The results of 16S rRNA sequencing are shown in Figures 3, 4. The good’s-coverage index showed that the depth of sequencing was adequate. The α-diversity was measured including community richness (Figures 3B,C) and diversity (Figure 3D). Although no significant differences were observed between the four groups, the trend of increase in the community richness was observed with the increase in fructose concentration. Through the Shannon species diversity index (Figure 3D), the diversity of fructose groups showed a decreasing trend as compared with the CON and 10% GLU groups.

The community structural changes were then analyzed using unsupervised multivariate statistical methods, including non-metric multidimensional scaling (NMDS) and PCA. As shown in Figure 3E, all four groups presented a distinct clustering of microbiota composition, and the fructose groups had a similar structure to that of the glucose group. Moreover, the comparison of heatmaps based on unweighted UniFrac distances (Figure 3F) showed lower distances between the CON and 10% GLU groups, compared with the fructose groups.

As shown in Figure 4A, analysis at the phylum demonstrated that extra glucose and fructose intake significantly decreased the relative abundance of *Firmicutes*, *Saccharibacteria*, and *Acidobacteria* while increasing the relative abundance of *Actinobacteria* and *Verrucomicrobia*, compared to the control group. Furthermore, the analysis at the genus level was similar to that at the phylum level (Figure 4B). Compared to the control group, extra glucose and fructose intake significantly decreased the relative abundance of *Lachnospiraceae* and increased the relative abundance of *Allobaculum*. Extra fructose intake decreased the relative abundance of *Ruminococcaceae* and increased the relative abundance of *Lactobacillus*. However, extra glucose intake increased the relative abundance of *Bacteroides*. Interestingly, the relative abundance of *Clostridium* was increased by extra fructose intake whereas it was decreased by extra glucose intake. LEfSe analysis (Supplementary Figure 1) further revealed that *Clostridia* and *Ruminococcaceae* were the two most abundant bacteria in the CON group. *Allobaculum* was the most abundant bacterium in the 10% FRU group, while *Lactobacillales* was the most abundant one in the 20% FRU group. The most abundant bacterium in the 10% GLU group was *Bacteroidaceae*.

### Excessive fructose intake influenced energy metabolism

**FIGURE 5:**
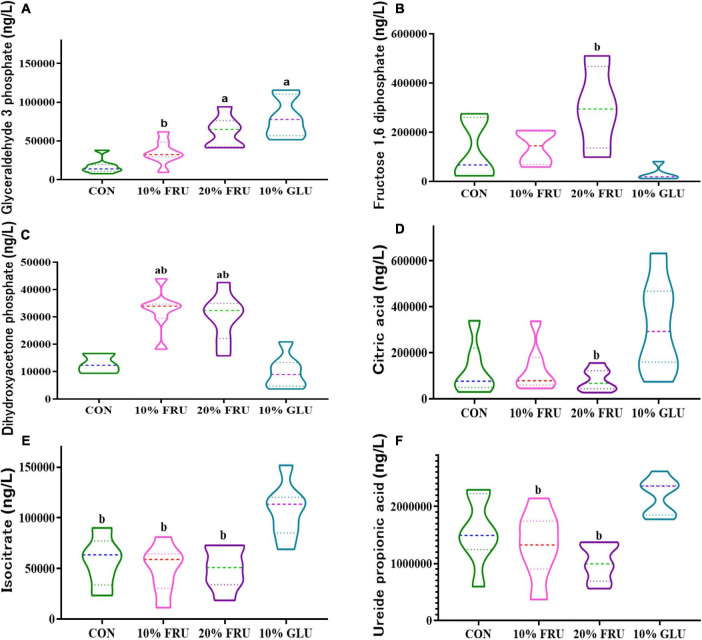
Effects of excessive fructose intake on the metabolites of central carbon metabolism in rats: **(A)** glyceraldehyde-3-phosphate, **(B)** fructose-1,6-diphosphate, **(C)** dihydroxyacetone phosphate, **(D)** citric acid, **(E)** isocitrate, and **(F)** ureide propionic acid. The values were presented as mean ± SD (*n* = 7); a means *p* < 0.05 compared with the CON group and b means *p* < 0.05 compared with the 10% GLU group.

KEGG analysis of the signaling pathways that the different bacteria act on indicated that changes in microbiota might affect energy metabolism (Supplementary Figure 2). Twenty-five metabolites related to the central carbon metabolism (CCM) pathway were detected, including glucose-involved tricarboxylic acid cycle (TCA), glycolysis pathway (EMP), and pentose phosphate pathway (PPP). Figure 5 shows the major differences in six metabolites. The level of glyceraldehyde-3-phosphate was higher in the FRU and GLU groups than that in the CON group. Fructose-1,6-diphosphate and dihydroxyacetone phosphate in FRU groups were not only higher than those of the CON group but also statistically higher than those of the 10% GLU group (*p* < 0.05). In contrast, isocitrate levels in the 10% GLU group were significantly higher than those of the CON and FRU groups. In addition, the level of citric acid was higher in the 10% GLU group than that of the 20% FRU group.

### Lower levels of bone formation and resorption were found after excessive fructose intake

**FIGURE 6:**
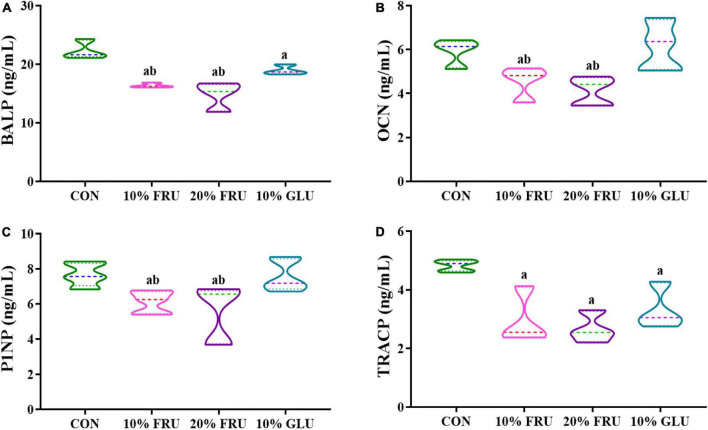
Analysis of serum markers of bone formation and resorption: **(A)** BALP, **(B)** OCN, **(C)** P1NP, and **(D)** TRACP. The values were presented as mean ± SD (*n* = 7); a means *p* < 0.05 compared with the CON group and b means *p* < 0.05 compared with the 10% GLU group.

Figure 6 shows bone metabolism markers levels in the different experimental groups. BALP, which peaked during the pre-osteoblast stage of differentiation, showed significantly lower levels in fructose and glucose groups when compared with the control group. In addition, BALP levels were even lower in FRU groups (10 and 20%) than those in the 10% GLU group. For the bone formation markers P1NP and OCN, the levels in FRU groups also were significantly lower than those of the CON and 10% GLU groups (*p* < 0.05), while no significant differences were found between the CON and 10% GLU groups. Moreover, TRACP, which was considered an indicator of bone absorption, was observed significantly lower in the GLU and FRU groups than that in the CON group (*p* < 0.05). In contrast, there were no significant differences between FRU and GLU groups.

## Discussion

Findings from previous studies investigating the associations between fructose intake and bone development were not consistent. The potential effects of fructose intake on the gut–bone axis in adolescent mammals also remained unclear. In this study, we investigated the effects of the oral intake of fructose for 10 weeks on the development of bone growth and GM. Our results suggest that excessive fructose intake may inhibit bone development in adolescence. The potential mechanism might be that excessive fructose intake affected the intestinal microbiota homeostasis in the body, thus changing the energy metabolism level, resulting in abnormal bone metabolism.

In adolescents, the consumption of sugar-sweetened beverages often leads to excessive fructose consumption (Wu et al., 2021). Therefore, we adopted the animal model of free drinking fructose solution to simulate the actual situation of adolescents. In mammals, the metabolic pathways and intermediates of fructose metabolism are significantly different from that of glucose. These differences could lead to unique metabolic effects. Intracellular glucose is first catabolized by glucokinase to glucose-6-phosphate and then enters the glycolysis pathway through a series of reactions. The conversion of fructose to fructose-1-phosphate is catalyzed by fructokinase and then directly decomposed into dihydroxyacetone phosphate and glyceraldehyde. Glyceraldehyde is phosphorylated to phosphoglyceraldehyde and that in turn can enter the tricarboxylic acid cycle to provide energy. Unlike glucokinase, fructokinase is not regulated by negative feedback from substrates (Sun and Empie, 2012). In case of excessive fructose intake, the energy metabolism changes and fat production speeds up, which would further trigger the development of metabolic syndrome (Faienza et al., 2020). This was supported by results from the current study. Compared to the control group, extra fructose intake could promote weight gain and then induce elevated blood glucose and abnormal lipid metabolism. Although the blood glucose and fructose levels showed no significant differences between the fructose and glucose groups when given at the same concentration, the blood HDL-C in the 10% FRU group showed decreasing trend as compared to the 10% GLU group.

The effects of fructose intake on bone development are different. Yarrow et al. showed that high fructose diets had a negative effect on the skeleton of 8-week-old male SD rats (Yarrow et al., 2016). Tian et al. observed that bone mass increased initially and then decreased in the 6–7-week-old mice with high fructose diet (Tian et al., 2018). The present results showed that rats in the 20% FRU group had lower BV/TV and Tb.N than the CON and 10% GLU groups, which indicated that excessive fructose intake could inhibit bone development, reducing the number of trabecular and bone mass. The higher Tb. Sp and Po(tot) also confirmed the effects of excessive fructose intake on bone development. Although there were no significant differences between the control, 10% GLU, and 10% FRU groups, the 10% FRU group showed a trend of poor bone development, while the 10% GLU group showed a favorable trend of bone development. It was speculated that the influences on bone development were not simply due to the energy provided by excessive carbohydrate intake.

The crosstalk between the GM and bone metabolism has been investigated in different physiological and pathological situations, including estrogen deficiency, aging, and obesity (Luo et al., 2015; Ma et al., 2020a,b). The GM is involved in the association between fructose intake and bone development, where excessive fructose intake may influence the amount and diversity of bacterial genera in the body. In this study, the dominant bacterial genera in the gut of rats were significantly different between experimental groups. The ratio of *Allobaculum* and *Lachnospiraceae* was significantly different between groups receiving carbohydrates and control group. *Allobaculum*, as an intestinal mucin degrader, was reported to increase in abundance on high-fat or high-sucrose diets, even in animal models of diabetes (Li et al., 2021). *Lachnospiraceae*, as a beneficial bacterium, can promote hematopoiesis and intestinal injury repair through the production of a large number of short-chain fatty acids (SCFAs) and metabolites of tryptophan (Guo et al., 2020). The low abundance of *Lachnospiraceae* and high abundance of *Allobaculum* suggested that the intestinal microbial dysbiosis occurred in rats fed with excessive fructose and glucose. It was interesting that significantly lower abundance of *Ruminococcaceae_9* and dose-dependent elevation in the abundance of *Lactobacillus* were observed in fructose groups, while no significant difference was found in the glucose group, compared with the control group. It was suggested that the effect of excessive fructose intake on bone development might be partly due to its influence on the GM. Several studies reported that vitamin K_2_, a core nutrient of bone metabolism, was mainly produced *via* GM, especially *Ruminococcaceae* (Karl et al., 2015). It was speculated that the influence of excessive fructose intake on bone development might partly be due to the decrease of vitamin K_2_ level produced *via Ruminococcaceae*. *Lactobacillus* reported some benefits for bone metabolism (Lu et al., 2021). However, this study showed higher abundance of *Lactobacillus* in fructose groups. Through the analysis of GM, it was found that fructose and glucose have different effects on GM, which might lead to their different effects on bone development.

To further explore the potential mechanism, pathway inference was performed through KEGG. As carbohydrates are the main nutrients for energy, energy metabolism was the main focus of this study. The change in energy metabolism indicated that the different compositions of GM in these groups might induce changes in energy metabolism. In this study, the increase of glyceraldehyde-3-phosphate in the fructose group and glucose group suggested that excessive carbohydrate intake did affect the energy metabolism in adolescent rats. However, the dihydroxyacetone phosphate level in the fructose group was significantly higher than that in the glucose group, while the citric acid and isocitrate were lower than that in the glucose group. Since citric acid and isocitrate are important metabolites in the tricarboxylic acid cycle, low levels of them in the fructose groups suggested that there were not a large amount of fructose metabolites entering the tricarboxylic acid cycle (Li Z. W. et al., 2022). It was speculated that excessive fructose intake could not only consume a lot of energy during metabolism but also its metabolites do not enter the tricarboxylic acid cycle to provide energy for the body. As bone metabolism is a very vigorous process of energy metabolism, the disturbance of energy metabolism caused by excessive fructose intake might be one of the reasons why fructose may affect bone development in our study.

The effects of energy metabolism changes in bone development were then analyzed by examining the levels of some bone metabolic markers in each group. BALP, P1NP, and OCN represent the processes of early osteogenic differentiation, collagen deposition, and calcium deposition mineralization, respectively (Shiau et al., 2020). All three indexes showed a trend of decrease in the fructose group, which indicated that the osteogenic differentiation activity of rats with excessive fructose intake decreased. TRACP reflects osteoclast activity to some extent and represents the condition of bone dissolution (Xu et al., 2020). Excessive fructose intake also showed decreased osteoclast activity. Energy metabolism plays an important role in the process of osteogenic and osteoclast differentiation, and both have strong energy requirements during the process of differentiation (Yang et al., 2020; Zheng et al., 2020). The reduction in bone formation and osteoclast differentiation induced by excessive fructose intake was consistent with the changes in energy metabolites detected in our study. It was hypothesized that excessive fructose intake leads to high energy requirements for metabolism thus leading to energy metabolism disorder in adolescence and inhibiting osteogenesis and osteoclast differentiation. Puberty is a period of vigorous osteogenic differentiation, and the inhibition of this process caused by insufficient energy production induced by excessive fructose intake seriously affected the development of bone, which resulted in weaker than in the normal control group. Bone development was better after glucose consumption than after fructose consumption, which was consistent with energy metabolism, indicating that excessive glucose intake mainly fueled the tricarboxylic acid cycle to provide energy for the body, without excessive negative impact on bone development.

In conclusion, excessive consumption of fructose may impair bone development in adolescent mammals. One of the possible underlying mechanisms is that fructose could change the GM of adolescent rats thus inducing the disturbance of energy metabolism, which influenced the balance of bone metabolism. Our findings indicate the significance of monitoring the intake of fructose in adolescents and provide novel perspectives for understanding the role of excessive fructose intake on bone development.

## Data availability statement

The raw sequencing data are deposited into the NCBI BioProject (http://www.ncbi.nlm.nih.gov/bioproject/841965) under accession number: PRJNA841965. The study protocol and the datasets generated during and/or analyzed during the current study will be available from the corresponding author on reasonable request.

## Ethics statement

The animal study was reviewed and approved by Animal Care and Use Committee of the Medical College, Qingdao University.

## Author contributions

TG used micro-CT to measure femur data and wrote the manuscript. CT carried out animal feeding and bone marker detection analysis. GT worked on the gut microbiome analysis. LM assisted in the bioactivity and energy metabolism analysis. LX and WL provided professional guidance in endocrinology and pediatrics. JC and FZ assisted in the analysis of body weight and glucolipid metabolism. HZ designed this experiment and revised the manuscript. AM guides the experiment. All authors contributed to the article and approved the submitted version.
