# Supplementary material for: Excessive fructose intake inhibits skeletal development in adolescent rats via gut microbiota and energy metabolism
Source: Front Microbiol. 2022 Sep 14;13:952892. doi: 10.3389/fmicb.2022.952892 (PMC9519145; doi:10.3389/fmicb.2022.952892)
Supplement: Supplementary file 1 [file Table_1.DOCX]

Supplementary Material

## Supplementary Figures


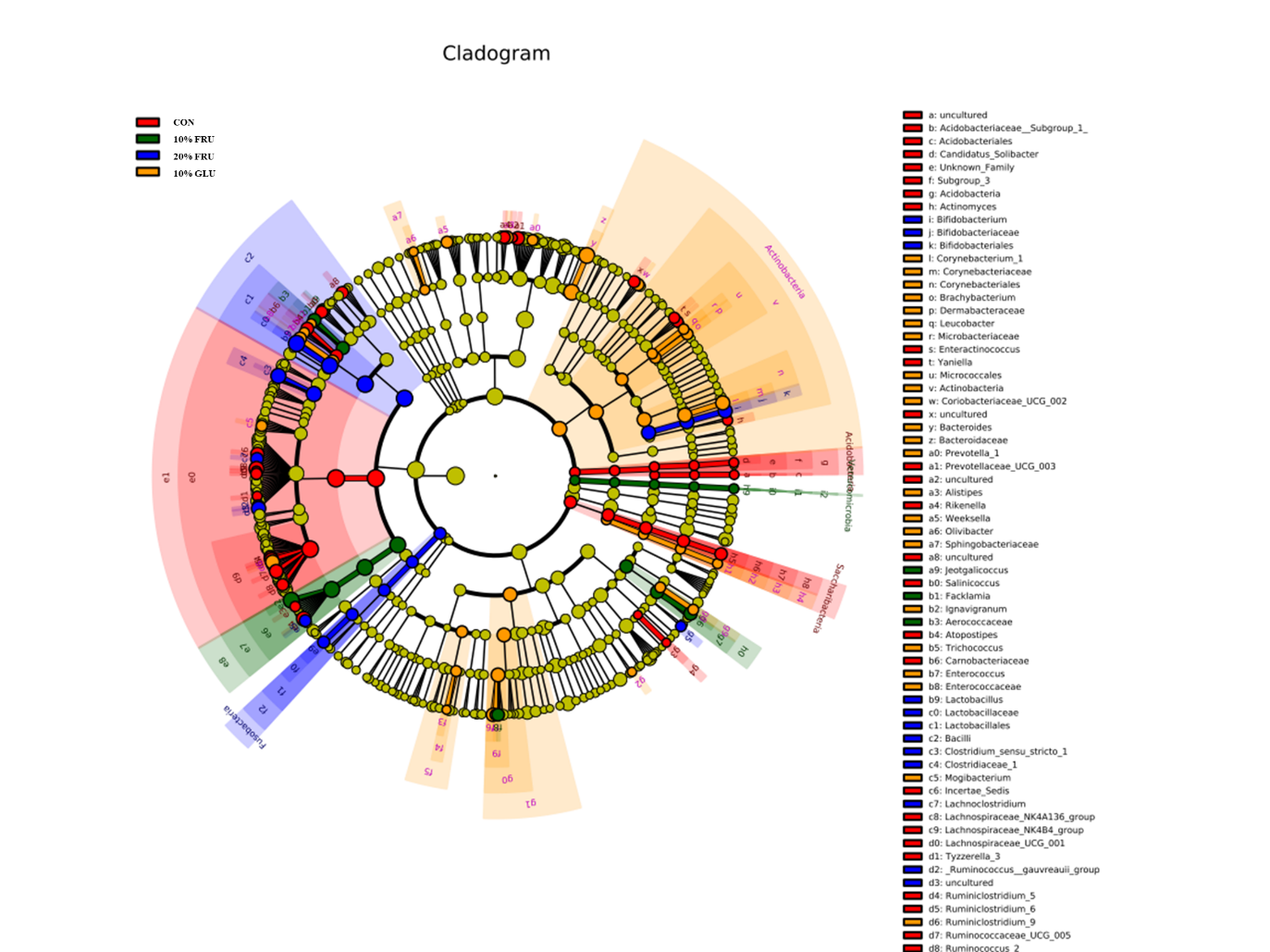


**Supplementary Figure 1.** LEfSe analysis of the gut microbiota at genus level.


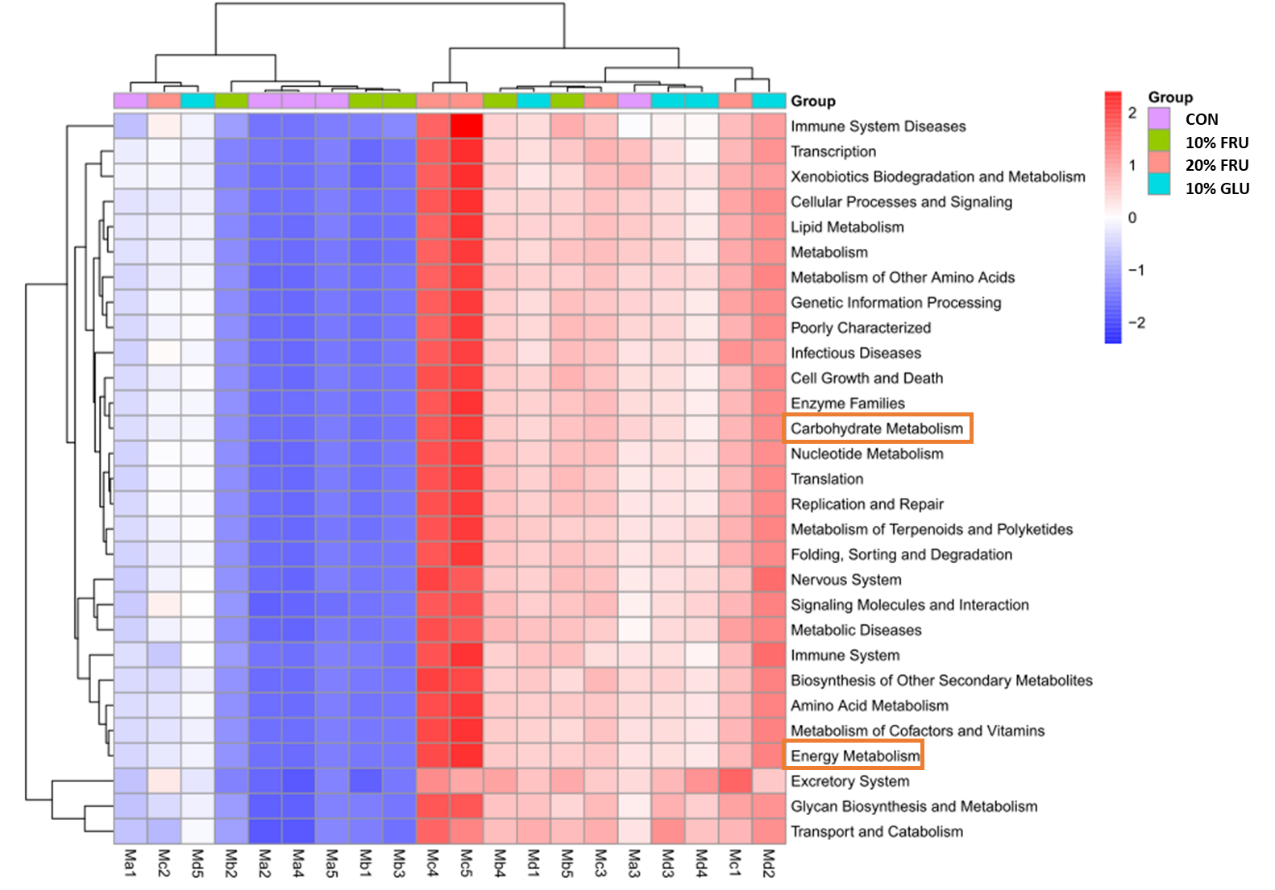


**Supplementary Figure 2.** Mechanism of inference based on gut microbiota via KEGG.
